# Olfactory dysfunction as an early predictor for post‐COVID condition at 1‐year follow‐up

**DOI:** 10.1002/brb3.3574

**Published:** 2024-06-06

**Authors:** Christoffer Granvik, Sara Andersson, Linus Andersson, Camilla Brorsson, Mattias Forsell, Clas Ahlm, Johan Normark, Alicia Edin

**Affiliations:** ^1^ Department of Clinical Microbiology Umeå University Umeå Sweden; ^2^ Department of Integrative Medical Biology Umeå University Umeå Sweden; ^3^ Department of Psychology Umeå University Umeå Sweden; ^4^ Department of Diagnostics and Intervention Umeå University Umeå Sweden

**Keywords:** COVID‐19, health‐related quality of life, long covid, olfactory dysfunction, post‐COVID condition (PCC)

## Abstract

**Background:**

Olfactory dysfunction together with neurological and cognitive symptoms are common after COVID‐19. We aimed to study whether performance on olfactory and neuropsychological tests following infection predict post‐COVID condition (PCC), persisting symptoms, and reduced health‐related quality of life.

**Methods:**

Both hospitalized (N = 10) and non‐hospitalized individuals (N = 56) were enrolled in this prospective cohort study. Participants were evaluated 1–3 months after infection with an olfactory threshold test and neuropsychological tests, which was used as predictors of PCC. A questionnaire outlining persisting symptoms and the validated instrument EuroQol five‐dimension five‐level for health‐related quality of life assessment were used as outcome data 1 year after infection (N = 59). Principal component analysis was used to identify relevant predictors for PCC at 1 year.

**Results:**

Objectively assessed olfactory dysfunction at 1–3 months post infection, but not subjective olfactory symptoms, predicted post‐COVID condition with reduced health‐related quality of life (PCC+) at 1 year. The PCC+ group scored more often below the cut off for mild cognitive impairment on the Montreal Cognitive Assessment (61.5% vs. 21.7%) and higher on the Multidimensional Fatigue Inventory‐20, compared to the group without PCC+.

**Conclusion:**

Our results indicate that objectively assessed, olfactory dysfunction is a predictor for PCC+. These findings underscore the importance of objective olfactory testing. We propose that olfactory screening in the early post‐acute phase of COVID‐19 infection might identify individuals that are at higher risk of developing long‐term health sequalae.

## Graphical abstract

Objectively assessed olfactory dysfunction at one to three months after COVID‐19, but not subjective olfactory symptoms, predicted post‐COVID condition (PCC) at one year. We propose that olfactory screening in the early post‐acute phase of COVID‐19 infection might identify individuals that are at higher risk of developing long‐term health sequalae.

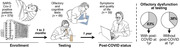

## 1 INTRODUCTION

A large and growing number of subjects globally report persisting symptoms after COVID‐19. Various terms have been used for the condition such as “long COVID”, “post‐acute sequelae of SARS‐CoV‐2 infection,” and “post‐acute COVID‐19 syndrome.” The term “post‐COVID condition” (PCC) was adopted by WHO in 2021 (WHO, 2021). According to modeled estimates, at least 6% of infected individuals develop PCC. This estimation predict that 45 million people suffer from PCC among the over 750 million confirmed COVID‐19 cases to date (Collaborators GBoDLC, 2022; WHO, 2024). Individuals with PCC experience a wide variety of symptoms indicating multiple underlying mechanisms and clinical phenotypes. Due to the high prevalence of PCC in published cohorts, we recently suggested adding reduced health‐related quality of life (HRQoL) to the definition to improve the identification of patients in need of clinical follow‐up and rehabilitation efforts (Ahmad et al., 2023).

Neurological and cognitive symptoms are frequently reported following acute COVID‐19 infection, especially in groups with reduced HRQoL. The most prevalent symptoms, according to a newly published systematic review conducted by Zeng et al. (2022), are cognitive deficits, memory impairment, and loss of taste or smell. Multiple studies also show that patients with PCC demonstrate reduced performance on tests that assess cognitive function (Becker et al., 2021; Mazza et al., 2021; Pihlaja et al., 2023; Rass et al., 2022). Most at risk are individuals who had a high severity of the initial infection (Becker et al., 2021; Mattioli et al., 2022; Pihlaja et al., 2023). However, cognitive impairment may persist also among individuals with initial mild COVID‐19 infection (Del Brutto et al., 2022; Pihlaja et al., 2023).

Olfactory dysfunctions, including hyposmia, anosmia, and parosmia, are common symptoms of COVID‐19 infection. When assessed with an odor identification task, 96% of study participants had measurable deficits in the acute phase of COVID‐19 (Moein et al., 2020). Most of these did improve over the first weeks after infection, but a minority experienced persisting olfactory deficits months and in some cases years after infection (Lechien et al., 2023). Olfactory dysfunction has also been associated with disease progression and severity. Anosmia has been associated with lower mortality and a lower admission rate to intensive care units in COVID‐19 patients (Talavera et al., 2020). Furthermore, individuals that experience persisting anosmia seem to be at higher risk of developing PCC (Cristillo et al., 2021; Thronicke et al., 2022; Xydakis et al., 2021). Methods to assess olfactory dysfunction are less standardized than those in for example, the visual and auditory domain. The association between subjective acuity and objective tests of olfactory function is low (Cavazzana et al., 2018). It has therefore been recommended that olfactory function should be established using psychophysical testing procedures that includes a threshold, and preferably also a supra‐threshold measure (Hummel et al., 2017).

We aimed to understand the impact of SARS‐CoV‐2 infection on the performance on olfactory and neuropsychological tests, and if such tests can identify individuals of risk for PCC, long‐term symptoms, and/or reduced HRQoL. Herein, we construct predictors for PCC outcomes at 1 year post infection using olfactory threshold screening data and neuropsychological testing in the sub‐acute phase of the disease.

## 2 METHODS

### 2.1 Study design and patient cohort

This study is part of the prospective, multicentre study, CoVUm (ClinicalTrials.gov Identifier: NCT04368013). The study protocol has been previously described (Ahmad et al., 2023). In brief, we enrolled hospitalized patients (≥18 years of age, N = 247) and non‐hospitalized (≥15 years of age N = 332) individuals with an SARS‐Cov‐2‐positive PCR test from April 2020 to June 2021. Individuals who were unable to provide informed consent or unable to read or communicate in Swedish were excluded. Data collected included baseline characteristics, disease severity, level of care, and clinical and laboratory parameters. The Charlson comorbidities index was used to quantify comorbidity‐based disease burden and mortality risk (Roffman et al., 2016). REDCap electronic data capture tools, hosted by Umeå University, were used to store study data (Harris et al., 2009). Data export was done on August 22, 2022.

**FIGURE 1:**
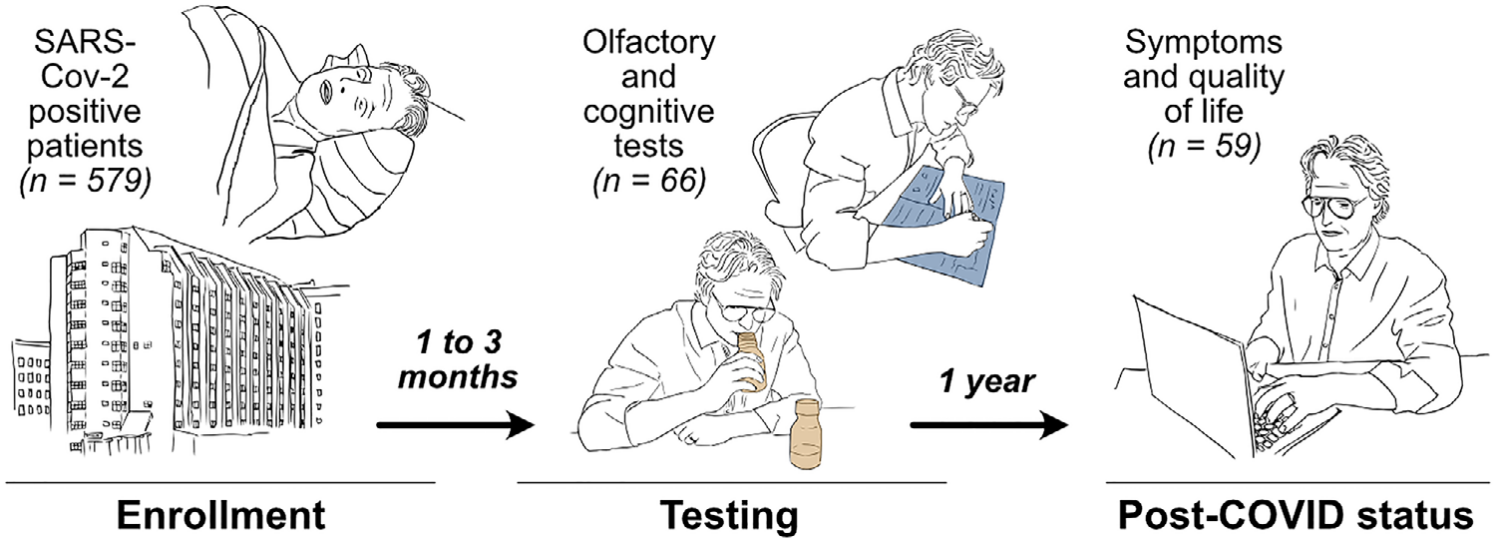
Graphical summary of the study design. *n*, number of individuals.

In this study, we subjected a sub‐cohort of study participants (N = 69) to cognitive assessment and olfactory testing 1–3 months after enrolment. The olfactory tests and cognitive assessment were performed separately by different study investigators. The study participants were selected using convenience sampling and examined between September and December 2020. The study design is summarized in Figure 1.

### 2.2 Predictors of post covid condition

#### 2.2.1 Olfactory assessment

A detailed description of the methods used for olfactory and cognitive assessment may be found in Supporting Information. We developed an olfactory screening method based on the validated and established Connecticut Chemosensory Clinical Research Center Threshold Test (Cain et al., 1988). We used the odorant *n*‐butanol as stimulus material (99.7% VWR) diluted in water to 3.5 mg/m^3^ and 0.04 mg/m^3^, corresponding to dilution steps 2 and 6 in Cain et al. (1988). The materials and procedures utilized have previously been reported to produce almost identical thresholds as those established through professional grade olfactometric procedures (Andersson et al., 2020).

The test comprised a threshold screening part where participants were presented with the stimuli in ascending order (i.e., first dilution step 6, then dilution step 2). The test also included a supra‐threshold assessment conducted after the threshold procedure. Participants were tasked to judge the intensity of all flasks (blank, dilution steps 6 and 2) using a Borg CR‐100 rating scale (Borg & Borg, 2002).

#### 2.2.2 Cognitive assessment

The Swedish version of the Montreal cognitive assessment (MoCA) was used as a brief screening tool for global cognitive function to detect mild cognitive impairment. To correct for education effects, one point was added to the total score for participants with 12 years of education or less (if total score < 30p), and <26p was used as a cut off for indicating mild cognitive impairment (Nasreddine et al., 2005).

Verbal learning and episodic memory were evaluated with the list learning test and the list recall test from the Repeatable Battery for the Assessment of Neuropsychological Status (Randolph et al., 1998). Scandinavian age‐corrected normative data were used to generate standardized outcome scores for verbal learning and episodic memory (Randolph, 2013).

Working memory was assessed with the letter‐number sequencing test and speed and attention were evaluated with the coding test from Weschler Adult Intelligence Scale IV (Wechsler, 2008). Scandinavian, age‐corrected norms were used to generate scaled scores with a mean of 10 and a standard deviation of 3 (Wechsler, 2010). The tests were administered by licenced neuropsychologists according to clinical regulations.

#### 2.2.3 Clinical scoring instruments

Symptoms of depression and anxiety were measured using the Hospital Anxiety and Depression Scale (Zigmond & Snaith, 1983). It consists of 14 items distributed on two subscales: anxiety and depression. Scores of 8p and above indicates symptoms consistent with clinically relevant levels of depression or anxiety.

Fatigue was measured with the Multidimensional Fatigue Inventory‐20 (Smets et al., 1995), a self‐report instrument that evaluates fatigue in five different dimensions: general fatigue, physical fatigue, reduced motivation, reduced activity and mental fatigue. Each dimension score from 4p to 20p, with a higher score indicating more fatigue.

The Karolinska Sleep Questionnaire was used to measure symptoms of sleep difficulties and sleepiness such as insomnia, wake‐ups, snoring and daytime sleepiness (Nordin et al., 2013). Index ≤3 was used as a cut off for indicating problems with a specific symptom.

### 2.3 Outcomes 1‐year post COVID‐19

A custom questionnaire including 15 symptoms was used to assess persistent symptoms at 1‐year post infection, as previously described (Ahmad et al., 2023). Self‐experienced dyspnoea was evaluated using the modified Medical Research Council scale (Nishiyama et al., 2010), and cut‐off for dyspnoea was set at score ≥1.

The EuroQol five‐dimension five‐level questionnaire (EQ‐5D‐5L) and the EuroQol Visual Analogue Scale (EQ‐VAS) were used to assess the patient's HRQoL after COVID‐19 infection. The results were converted to an index value (EQ‐5D Index) by using United Kingdom as the reference population (https://euroqol.org/eq‐5d‐instruments/eq‐5d‐5l‐about/).

Post‐COVID condition with reduced HRQoL, PCC+ (previously PACS+) was defined, as previously described, as the prevalence of ≥1 symptom at follow‐up, together with either moderate (score ≥3) difficulties in two or more dimensions of EQ‐5D‐5L and/or self‐assessed overall health ≤60 in EQ‐VAS (Ahmad et al., 2023). Individuals in the cohort who did not meet the criteria for PCC+ were designated as PCC−.

### 2.4 Statistical analysis

Statistical analysis was performed using Jamovi (version 2.3.18) and R (version 4.2.2). Groups were compared using Mann–Whitney *U*‐test for continuous variables and *X* ^2^‐test or Fisher's exact test for categorical variables. Correlation between variables was analysed using Spearman's rank correlation and controlled for age, gender, hospitalization, and Charlson comorbidity index.

Multivariable logistic regression was performed in R in order to explore predictive variables for the development of PCC+. Variable reduction was performed using principal component analysis (PCA) including variables from olfactory detection threshold test and cognitive assessments. The first two components were used as variables for the predictive regression model. The analysis was adjusted for confounders age, sex, Charlson comorbidity index, and hospitalization. The results are presented as odds ratios (ORs) with 95% confidence intervals. Significance level was set at the 5% level (*p*‐value < 0.05). No correction for multiple testing was applied due to the exploratory nature of this study. Missing values were imputed with the missMDA package (Josse & Husson, 2016).

## 3 RESULTS

### 3.1 Study cohort

At data export, there were 579 individuals present in the CoVUm database out of which 431 attended the 1‐year follow‐up visit. Out of the 69 individuals that underwent olfactory testing and neuropsychological assessment, 66 (96%) attended the 1‐year follow‐up visit and 59 (86%) completed the EQ‐5D‐5L questionnaire (Figure S1). The median time for examination was 45 days after disease onset (interquartile range [IQR] 37.0–99.3). For the exact number of included participants in each individual test and analysis, we refer to the information under each subheading.

The total cohort consisted of 26 of 66 women (39%) and the median age was 51.5 years (47.0–59.8) (Table 1). Median body mass index (BMI) was significantly higher in hospitalized compared to non‐hospitalized individuals (28.5 vs. 25.4, *p* = 0.033) (Table S1). When comparing baseline characteristics of study participants with PCC+ and PCC−, two significant differences were observed. Individuals with PCC+ had higher median BMI (28.9 vs. 25.1, *p* < 0.001) and were more likely to have hypertension (62% vs. 11%, *p* < 0.001) (Table S2). No significant differences were found between genders.

**TABLE 1:** Demographic and baseline characteristics of the study cohort, divided by sex.

	Total (*n* = 66)	Women (*n* = 26)	Men (*n* = 40)	*p*‐value
Age in years—median (IQR)	51.5 (47.0–59.8)	55 (47.3–60.0)	51 (46.5–59.0)	0.491^ª^
BMI—median (IQR)	25.6 (23.8–27.4)	25.2 (24.3–26.7)	26.3 (23.4–27.5)	0.577^ª^
Comorbidities—*n* (%)				
Diabetes	4 (6.0)	1 (3.8)	3 (7.5)	1.000^b^
Hypertension	15 (22.7)	7 (26.9)	8 (20.0)	0.558^b^
Cardiovascular disease^c^	5 (7.6)	1 (3.8)	4 (10.0)	0.641^b^
Chronic lung disease^d^	11 (16.7)	6 (23.1)	5 (12.5)	0.319^b^
Asthma	10 (15.2)	6 (23.1)	4 (10.0)	0.175^b^
Autoimmune disease^e^	5 (7.6)	3 (11.5)	2 (5.0)	0.375^b^
Immunocompromised^f^	1 (1.5)	0 (0.0)	1 (2.5)	1.000^b^
Malignancy^g^	1 (1.5)	1 (3.8)	0 (0.0)	0.394^b^
Brain injury^h^	5 (7.6)	1 (3.8)	4 (10.0)	0.641^b^
Psychiatric illness^i^	6 (9.1)	3 (11.5)	3 (7.5)	0.673^b^
Dyslexia	3 (4.5)	1 (3.8)	2 (5.0)	1.000^b^
Chronic sinuitis	1 (1.5)	0 (0.0)	1 (2.5)	1.000^b^
CCI—median (IQR)	0 (0–0)	0 (0–0)	0 (0–0)	0.609^ª^
Smoking status—*n* (%)^j^				0.859^k^
Non‐smoker	54 (84.4)	20 (83.3)	34 (85.0)	
Current smoker	0 (0.0)	0 (0.0)	0 (0)	
Former smoker	10 (15.6)	4 (16.7)	6 (15.0)	
Level of education—*n* (%)^l^				0.575^k^
Lower	0 (0.0)	0 (0.0)	0 (0.0)	
Medium	23 (34.8)	8 (30.8)	15 (37.5)	
Higher	43 (65.1)	18 (69.2)	25 (62.5)	
Hospitalized—*n* (%)	10 (15.1)	5 (19.2)	5 (12.5)	0.498^b^
Other first language—*n* (%)	7 (10.6)	3 (11.6)	4 (10.0)	1.000^b^

Abbreviations: BMI, body mass index; CCI, Charlson comorbidities index; IQR, interquartile range; *n*, number of patients.

^ª^ Mann–Whitney *U* test.

^b^ Fischer's exact test.

^c^ Ischemic heart disease, congestive heart failure, arrythmias, aortic disease, valvular heart disease, or peripheral arterial insufficiency.

^d^ Chronic obstructive pulmonary disease and asthma.

^e^ Including rheumatic diseases.

^f^ Immune deficiency diseases or immunosuppressive/immunomodulatory medication.

^g^ Solid localized tumor, lymphoma, or leukemia.

^h^ Previous brain surgery or head trauma.

^i^ History of anxiety, depression, or exhaustion syndrome.

^j^ Smoking status is missing in two patients. The analysis is based on 64 patients.

^k^ *X* ^2^‐test.

^l^ Lower: Less than 3 years beyond Swedish compulsory school. Medium: Three years beyond Swedish compulsory school, but no college or university degree. Higher: University or college degree.

### 3.2 High prevalence of symptoms and reduced health‐related quality of life 1 year after infection

At the 1‐year follow‐up visit, 45 (68.2%) of the 66 individuals that completed neuropsychological assessment and olfactory testing reported at least one symptom. Most common were concentration difficulties (32%), physical fatigue (32%), and neurological symptoms such as impaired memory function (35%), hyposmia (32%), difficulties finding words (32%), and mental fatigue (30%) (Table 2).

**TABLE 2:** Summary of symptoms reported by the participants at 1‐year follow‐up, divided by sex.

	Total (*n* = 66)	Women (*n* = 26)	Men (*n* = 40)	*p*‐value
At least one symptom—*n* (%)	45 (68.2)	21 (80.8)	24 (60.0)	0.106
Respiratory symptoms—*n* (%)	15 (22.7)	9 (34.6)	6 (15.0)	0.078
Cough	10 (15.2)	4 (15.4)	6 (15.0)	1.000
Dyspnea (mMRC > 1)	10 (15.2)	8 (30.8)	2 (5.0)	**0.010**
Neurological symptoms—*n* (%)	41 (62.1)	21 (80.8)	20 (50.0)	**0.019**
Dizziness	8 (12.1)	6 (23.1)	2 (5.0)	0.050
Headache	16 (24.2)	8 (30.8)	8 (20.0)	0.384
Hyposmia/dysgeusia	21 (31.8)	13 (50.0)	8 (20.0)	**0.015**
Impaired memory function	23 (34.8)	12 (46.2)	11 (27.5)	0.186
Difficulties finding words	21 (31.8)	12 (46.2)	9 (22.5)	0.060
Mental fatigue	20 (30.3)	11 (42.3)	9 (22.5)	0.106
Psychiatric symptoms—*n* (%)	31 (47.0)	13 (50.0)	18 (45.0)	0.802
Panic attacks	6 (9.1)	4 (15.4)	2 (5.0)	0.202
Concentration difficulties	21 (31.8)	10 (38.5)	11 (27.5)	0.422
Sleeping difficulties	16 (24.2)	6 (23.1)	10 (25.0)	1.000
Nightmares	4 (6.1)	3 (11.5)	1 (2.5)	0.292
Other—*n* (%)	31 (47.0)	15 (57.7)	16 (40.0)	0.209
Myalgia	12 (18.2)	8 (30.8)	4 (10.0)	0.050
Physical fatigue	21 (31.8)	10 (38.5)	11 (27.5)	0.422
Restless legs	16 (24.2)	8 (30.8)	8 (20.0)	0.384
Upset stomach	8 (12.1)	6 (23.1)	2 (5.0)	0.050

*Note*: Significant *p*‐values (<0.05) are given in bold.

Abbreviation: *n*, number of patients. mMRC, modified Medical Research Council scale.

Among the 59 individuals that completed the EQ‐5D‐5L questionnaire, 13 (22.0%) fulfilled the PCC+ criteria. The most frequently reported problem at the 1‐year follow‐up was pain or discomfort (28.8%), followed by problems with usual activities (11.9%) and mobility (10.2%) (Table S3). A large proportion of individuals (18.6%) assessed their overall health to 60 or below at the EQ‐VAS, indicating low HRQoL. Five (8.5%) individuals <65 years reported a reduction in their ability to work of ≥25% at the 1‐year follow‐up, and four of those had PCC+.

### 3.3 Olfactory dysfunction in the early post‐acute phase of COVID‐19 infection predicts PCC+ after 1 year

Among the 66 individuals that completed the olfactory detection threshold test, anosmia was found in four (6.1%) individuals, 21 (31.8%) had hyposmia, and 41 (62.1%) individuals exhibited a normal sense of smell. Individuals belonging to the PCC+ group displayed a significantly higher degree of olfactory dysfunction (37.5% vs. 62.5%, *p* = 0.026) as compared to those in the PCC− group. Further, as seen in Table 3, individuals in the PCC+ group rated both *n*‐butanol dilutions (six and two) as significantly less intense than the PCC− group.

**TABLE 3:** Summary of the results from the neuropsychological and olfactory tests performed at 1–3 months after onset of COVID‐19. The analysis is based on participants’ post‐covid condition status at the 1‐year follow‐up visit. Post‐COVID condition+ (PCC+) was defined as at least one persisting symptom and reduced health‐related quality of life.

	Total (*n* = 59)	PCC+ (*n* = 13)	PCC− (*n* = 46)	*p*‐value
Odor detection threshold test, hyposmia or anosmia—*n* (%)	24 (40.7)	9 (69.2)	15 (32.6)	**0.026** ^a^
Borg scale—median (IQR)				
Control—median (IQR)	0 (0–2.5)	0 (0–0)	0 (0–4.50)	0.115^b^
Diluted 1:6—median (IQR)	5.0 (1.0–13.0)	1.0 (0.0–3.0)	8.5 (2.5–13.0)	**0.003** ^b^
Diluted 1:2—median (IQR)	25.0 (19.5–46.5)	10.0 (5.0–24.0)	30.0 (23.0–49.5)	**<0.001** ^b^
Montreal Cognitive Assessment—raw scores				
Total score—median (IQR)	27 (25.0–28.0)	25 (23.0–27.0)	26.0 (26.0–27.0)	0.091^b^
Less than 26 points—*n* (%)	18 (30.5)	8 (61.5)	10 (21.7)	**0.014** ^a^
Multidimensional fatigue inventory‐20^c^				
General fatigue—median (IQR)	14 (10.0–16.0)	16 (14–17.5)	13 (10–15)	**0.036** ^b^
Physical fatigue—median (IQR)	11 (7.0–16.0)	16 (15–17.5)	10 (7–14)	**0.001** ^b^
Reduced activity—median (IQR)	10 (7.0–12.5)	14 (12–17.5)	9 (7–12)	**<0.001** ^b^
Reduced motivation—median (IQR)	10 (7.0–12.5)	14 (10–15)	9 (7–11)	**0.002** ^b^
Mental fatigue—median (IQR)	12 (9.0–14.0)	14 (13.5–15.5)	12 (9–13)	**0.011** ^b^
Repeatable Battery for the Assessment of Neuropsychological Status—*z*‐score				
List learning test—median (IQR)^d^	−0.4 (−1.1 to 0.3)	−0.9 (−1.6 to −0.3)	−0.2 (−1.0 to 0.4)	0.111^b^
List recall test—median (IQR)^e^	−0.0 (−1.2 to 0.6)	−0.48 (−1.4 to 0.3)	0.0 (−1.0 to 0.6)	0.152^b^
Weschler Adult Intelligence Scale IV—scaled scores				
Letter‐number sequencing test—median (IQR)^f^	10.0 (9.0–10.0)	9 (8.0–10.0)	10 (9.0–10.3)	0.058^b^
Coding test—median (IQR)^d^	10.0 (9.0–11.0)	10 (8.5–11.5)	10 (9.0–11.0)	0.901^b^
Karolinska Sleep Questionnaire				
Insomnia—median (IQR)^g^	16.0 (12.0–19.0)	15.0 (10.0–18.3)	17.0 (13.0–20.0)	0.255^b^
Wake ups—median (IQR)^c^	13.0 (11.0–15.0)	11.5 (8.5–13.5)	13.5 (11.0–15.0)	0.110^b^
Snoring—median (IQR)^h^	16.0 (14.5–17.0)	16.0 (13.0–17.3)	17.0 (15.0–17.0)	0.449^b^
Sleepiness—median (IQR)^h^	26.0 (23.0–27.0)	23.0 (19.0–25.5)	26.0 (23.8–27.0)	0.051^b^
Hospital Anxiety and Depression Scale				
Depression—median (IQR)^c^	3.0 (1.0–5.0)	5.5 (4.8–7.3)	2.0 (1.0–4.0)	**0.003** ^b^
Anxiety—median (IQR)^c^	5.0 (2.0–8.0)	4.5 (2.0–8.8)	5.0 (2.0–7.3)	0.984^b^

*Note* ^:^ Significant *p*‐values (<0.05) are given in bold.

**Abbreviations**: IQR, interquartile range.; *n*, number of patients; PCC−, not post‐COVID‐19 condition+; PCC+, post‐COVID‐19 condition+.

^a^ Fischer's exact test.

^b^ Mann–Whitney *U* test.

^c^ The analysis is based on 56 patients.

^d^ The analysis is based on 52 patients.

^e^ The analysis is based on 51 patients.

^f^ The analysis is based on 49 patients.

^g^ The analysis is based on 57 patients.

^h^ The analysis is based on 55 patients.

**FIGURE 2:**
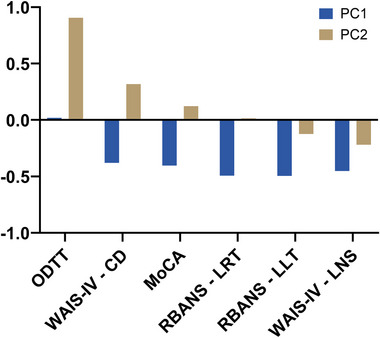
The graph represents loadings for principal components 1 and 2 from the principal component analysis of neuropsychological and olfactory tests. Loadings are the covariance between the variables of the analysis (here the results from the neuropsychological and olfactory tests) and the scaled components. A large loading (positive or negative) indicates a strong relationship between the variable and the component. MoCA, Montreal Cognitive Assessment; ODTT, olfactory detection threshold test; PC1, principal component 1; PC2, principal component 2; RBANS–list recall test, list recall test from the Repeatable Battery for the Assessment of Neuropsychological Status; RBANS–list learning test, list learning test from the Repeatable Battery for the Assessment of Neuropsychological Status; WAIS‐IV Coding test, coding test from Weschler Adult Intelligence Scale IV; WAIS‐IV letter‐number sequencing test, letter‐number sequencing test from Weschler Adult Intelligence Scale IV.

In order to predict PCC+ after 1 year, we constructed a new predictive variable with PCA using the results from the olfactory detection threshold test and cognitive assessment at the first timepoint. A total of 56 individuals were included, and three individuals were excluded due to high percentage of missing values, resulting in a total of 6.0% missing values. The cumulative proportion of variance for the first three components was 0.78. Score values for principal component 1 and 2 was used as variables to predict PCC+. Principal component two was primarily defined by olfactory detection threshold test and significantly predicted PCC+ with an adjusted OR of 2.93 (95% confidence interval 1.25–6.87, *p* = 0.014) in logistic regression analysis (Table 4). Important variables in principal component one were as follows: list learning test, list recall test, MoCA, letter‐number sequencing test, and coding test, but the result was not significant. The loadings of each component from the PCA are presented in Figure 2, and the contribution of each variable is presented in Figure S2. In summary, olfactory detection threshold test was most relevant in the PCA analysis and predicted PCC+ using logistic regression analysis.

**TABLE 4:** Results from logistic regression performed to assess association between test results of neuropsychological and olfactory tests, with principal components 1 and 2 from the principal component analysis.

	Adjusted odds ratio	*p*‐value
Principal component 1	0.61 (0.36–1.03)	0.063
Principal component 2	2.93 (1.25–6.87)	**0.014**
Age	1.07 (0.99–1.16)	0.085
Sex	6.02 (0.75–48.46)	0.092
Hospitalization	3.00 (0.82–20.46)	0.262
Charlson comorbidities index	3.71 (0.36–16.90)	0.090

*Note*: The results are presented as adjusted odds ratios with 95% confidence intervals. Significant *p*‐values (<0.05) are given in bold. The logistic regression analysis was adjusted for age, sex, Charlson comorbidity index, and hospitalization during acute COVID‐19.

Given the association between olfactory dysfunction and PCC+, we further explored the association between the rated intensity of *n*‐butanol and outcomes of relevance for post‐COVID status. We used the intensities for dilution step 6, as dilution step 2 may be slightly pungent (i.e., mediated by the trigeminal cranial nerve) for some individuals (ECETOC (European Centre for Ecotoxicology and Toxicology of Chemicals), 2003). Our results showed that the rated intensity had a significant, negative correlation with PCC+, persisting symptoms and a significant, positive correlation with EQ‐5D Index after 1 year. However, self‐rated olfactory functioning was not correlated with these outcomes (Table 5). Since time from infection to olfactory testing varied between subjects, we performed a correlation analysis between PCC+ status and number of days from infection to olfactory testing to exclude this as confounding factor. No significant correlation was found (data not shown).

**TABLE 5:** Summary of results of the Spearman's correlation analysis of olfactory tests after COVID‐19 infection and outcomes after 1 year.

		BORG Dilution step 6	Subjective odor deficit
Outcomes	Post‐COVID‐19 condition +	**−0.304 (0.024)**	0.131 (0.341)
	At least one symptom	**−0.320 (0.011)**	0.112 (0.338)
	Respiratory symptoms	0.009 (0.944)	0.013 (0.922)
	Neurological symptoms	**−0.351 (0.005)**	0.230 (0.072)
	Psychiatric symptoms	**−0.280 (0.027)**	−0.121 (0.349)
	Other symptoms	−0.199 (0.121)	−0.065 (0.614)
	EQ‐5D index	**0.426 (0.001)**	−0.018 (0.898)

*Note*: The analysis was adjusted for age, sex, Charlson comorbidity index, and hospitalization during acute COVID‐19. The results are presented as spearman's rho (*p*‐value). Significant *p*‐values (<0.05) are given in bold.

Abbreviation: EQ‐5D Index, EuroQol five‐dimension index.

### 3.4 Cognitive differences comparing PCC+ to PCC− at baseline

The median scores for the list learning test and the list recall test, the letter‐number sequencing test, and the coding test were all above −1.0 SD in both groups, indicating that learning, episodic memory, working memory, and speed/attention were within normal range at baseline. The PCC+ group scored lower in all tests except the coding test, but the difference was not significant (Table 3). Individuals with high level of education performed better in the list learning test (*p* = 0.048), the letter‐number sequencing test (*p* < 0.001), and the coding test (*p* = 0.001), compared to participants with medium level of education (Figure S3).

Out of 59 individuals with HRQoL data at follow‐up, 18 (27.3%) achieved a test score below the cut off indicating mild cognitive impairment on the MoCA test. A test score < 26p was significantly more common among the PCC+ group (61.5% vs. 12.2%, *p* = 0.014) (Table 3). The difference between the PCC+ and PCC− group remained when individuals with previous brain injury, dyslexia, psychiatric illness, and other first languages were excluded (N = 42, 83.3% vs. 19.4%, *p* = 0.005).

### 3.5 Fatigue was significantly more common in the PCC+ group early in the post‐acute phase

Individuals with PCC+ at the 1‐year follow up scored significantly higher in all dimensions in Multidimensional Fatigue Inventory‐20 compared to PCC− individuals (Table 3). The highest median results were found in the dimensions general fatigue (16 vs. 13, *p* = 0.036) and physical fatigue (16 vs. 10, *p* = 0.001). Also, the test dimensions mental fatigue (14 vs. 12, *p* = 0.011), reduced activity (14 vs. 9, *p* < 0.001), and reduced motivation (14 vs. 9, *p* = 0.002) showed high scores and the difference between groups was significant.

Fifty‐six study participants completed the Hospital Anxiety and Depression Scale. Fifteen of these (26.8%) scored eight points or higher on the subscale for anxiety and six (10.7%) individuals on the subscale for depression. Individuals with PCC+ had a significantly higher score on the subscale for depression (5.5 vs. 2.0, *p* = 0.003), but only three (25.0%) individuals had a score of eight or above. There was no significant difference between the results for PCC+ and PCC− on the anxiety subscale. Neither were there any significant differences in symptoms of sleep difficulties and sleepiness.

## 4 DISCUSSION

In this study, we investigated whether performance on olfactory and neuropsychological tests following COVID‐19 infection can predict PCC status, persisting symptoms, and reduced HRQoL in a prospective cohort comprising hospitalized and non‐hospitalized individuals.

A large part of individuals in the cohort (37.9%) reported olfactory dysfunction at baseline and almost one‐third at the 1‐year follow‐up, which is slightly higher than previously reported proportions (Lechien et al., 2023; Zeng et al., 2022). However, self‐reported olfactory acuity is unreliable (Cavazzana et al., 2018), which prompted us to also assess this by using a standardized olfactory screening test. The olfactory detection threshold test was the strongest predictive variable for PCC+ in our cohort, which lends credibility to the hypothesis that early olfactory dysfunction is associated with future PCC (Xydakis et al., 2021). An explorative follow‐up analysis further revealed that the reported intensity of the olfactory stimulus was associated with several lingering problems at the 1‐year follow‐up, including PCC status, neurological and psychiatric symptoms, and lower quality of life. Interestingly, self‐reported olfactory deficits were not associated with any of these outcomes.

Suggested mechanisms of COVID‐related smell loss include downregulated receptor proteins within olfactory receptor cells, blockage of receptors due to local inflammation, and damage to the olfactory mucosa, the olfactory bulb, or other central brain structures (Doty, 2022). Neuroinflammation has been proposed as a possible mechanism since patients with cognitive symptoms have been shown to have higher risk for olfactory dysfunction following COVID‐19 (Di Stadio et al., 2022). Early fears of direct central nervous system (CNS) damage caused by viral invasion through the olfactory pathway in patients with mild COVID‐19 is now deemed unlikely (Matschke et al., 2020). Our results do not support that the experience of sensory loss in itself explains reduced HRQoL and other persistent symptoms. The individuals who self‐reported olfactory deficits did not report lower HRQoL or other PCC symptoms at a higher frequency at follow‐up, which suggests that the experience of having an impaired sense of smell does not in itself predict future symptoms. Instead, our results support a theory where loss of smell could be an effect of (yet to be determined) an underlying factor that also perpetuates PCC symptoms. Olfactory deficits are regularly found to predict adversity and mortality. Although mechanisms are still largely unknown, a reoccurring argument is that olfaction is a fragile sense. Its integrity is reliant on well‐functioning systemic regenerative and homeostatic capabilities, and may thus constitute an index of general health (Van Regemorter et al., 2020). Immunological dysfunction has been observed in individuals with PCC as long as 8 months after infection (Phetsouphanh et al., 2022). Hypotheses regarding over‐reactive immune responses, autoimmunity, and persistent inflammation (Franke et al., 2023; Mehandru & Merad, 2022) may therefore be a possible explanation also for the observed olfactory deficits.

Cognitive impairment after COVID‐19 has been reported in multiple studies (Evans et al., 2021). We found that PCC+ individuals, compared with the PCC− group, expressed a mild global cognitive impairment at baseline, comparable to previous reports (Evans et al., 2021; Pihlaja et al., 2023) The neuropsychological tests did not predict PCC status. However, the PCC+ group scored below average in working memory, verbal learning and episodic memory and working memory deficits were at a trend level which might prove to be significant differences in a larger cohort. Fatigue was a frequently reported symptom in our cohort and the PCC+ group reported significantly higher general, physical, and mental fatigue than the group with PCC−. The high proportion of individuals with fatigue is in congruence with previous results (Ceban et al., 2022). Problems with fatigue in the early post‐acute phase are in our study associated with increased risk of long‐term symptoms and reduced HRQoL. Among symptoms of general, physical, and mental fatigue, our data also indicate a higher prevalence of depressiveness in the PCC+ group at the early post‐infectious phase, as compared to PCC−, although at a mild symptomatic level. Fatigue, depressiveness, and mild cognitive impairment together with olfactory deficits in the PCC+ group might reflect the presence of a prolonged systemic inflammation and/or autoimmune reaction in the CNS previously reported in PCC (Franke et al., 2023; Van Regemorter et al., 2020).

The strength of the present study is the combination of several validated standardized methods to assess olfactory dysfunction, cognitive impairment, and HRQoL following COVID‐19.

This study is limited by a small cohort size. Multiple trends could be observed that were not statistically significant due to lack of statistical power. This also made subgroup analysis difficult. Another limitation is the lack of pre‐COVID results from cognitive tests, which resulted in a lack of data on premorbid cognitive function. The level of education in this cohort was high, which could have influenced the results of the neurocognitive tests. Finally, repeated follow‐up olfactory and cognitive assessment would further have improved the understanding of the dynamics of symptoms and impairments in PCC+ individuals.

## 5 CONCLUSION

In summary, we present results from a prospective cohort with high‐resolution characterization of study participants based on objective and standardized tests. Our findings reveal an association between olfactory dysfunction in an early post‐acute phase after COVID‐19 infection and PCC‐related outcomes after 1 year. A rapid olfactory screening test, but not subjective smell loss predicted reduced HRQoL and persistent symptoms. We propose that an olfactory screening test, like the one used here, in the early postacute phase of COVID‐19 infection, may be used to identify individuals that are at higher risk of developing long‐lasting symptoms and reduced HRQoL after COVID‐19. Larger cohort studies are necessary to validate olfactory screening tests as prognostication methods for PCC.

## AUTHOR CONTRIBUTIONS

**Christoffer Granvik**: Writing—original draft; writing—review and editing; formal analysis; data curation; visualization. **Sara Andersson**: Conceptualization; investigation; methodology; validation; data curation; writing—review and editing. **Linus Andersson**: Conceptualization; methodology; investigation; validation; writing—review and editing; visualization; resources. **Camilla Brorsson**: Conceptualization; methodology; writing—review and editing. **Mattias Forsell**: Funding acquisition; writing—review and editing. **Clas Ahlm**: Funding acquisition; project administration; writing—review and editing. **Johan Normark**: Conceptualization; methodology; supervision; resources; project administration; funding acquisition; writing—review and editing. **Alicia Edin**: Investigation; writing—review and editing; project administration; data curation; supervision; validation.

## CONFLICT OF INTEREST STATEMENT

The authors declare that the research was conducted in the absence of any commercial or financial relationships that could be construed as a potential conflict of interest.

### PEER REVIEW

The peer review history for this article is available at https://publons.com/publon/10.1002/brb3.3574

## Supporting information

Supplementary Figure 1 Flowchart of participants included in the study cohort. Abbreviations: n, number of patients; EQ‐5D‐5L, EuroQol 5‐dimension 5‐level questionnaire.

Supplementary Figure 2 The graph represents percentage of contribution for variables in principal component 1 and 2 from the principal component analysis of all neuropsychological and olfactory tests. Abbreviations: PC1, Principal Component 1; PC2, Principal Component 2; ODTT, Olfactory Detection Threshold Test; WAIS‐IV CD, Coding Test from Weschler Adult Intelligence Scale IV; MoCA, Montreal Cognitive Assessment; RBANS—LRT, List recall test from the Repeatable Battery for the Assessment of Neuropsychological Status; RBANS—LLT, List learning test from the Repeatable Battery for the Assessment of Neuropsychological Status; WAIS‐IV LNS, Letter‐Number Sequencing Test from Weschler Adult Intelligence Scale IV.

Supplementary Figure 3 Results from neuropsychological assessment, divided by level of education. **Abbreviations**: RBANS—LLT, List learning test from the Repeatable Battery for the Assessment of Neuropsychological Status; RBANS—LRT, List recall test from the Repeatable Battery for the Assessment of Neuropsychological Status; WAIS‐IV LNS, Letter‐Number Sequencing Test from Weschler Adult Intelligence Scale IV; WAIS‐IV CD, Coding Test from Weschler Adult Intelligence Scale IV.

Supplementary information

## Data Availability

The data that support the findings of this study are available on request from the corresponding author. The data are not publicly available due to privacy or ethical restrictions.
