# Supplementary figures and images for: Olfactory dysfunction as an early predictor for post‐COVID condition at 1‐year follow‐up
Source: Brain Behav. 2024 Jun 6;14(6):e3574. doi: 10.1002/brb3.3574 (PMC11154814; doi:10.1002/brb3.3574)

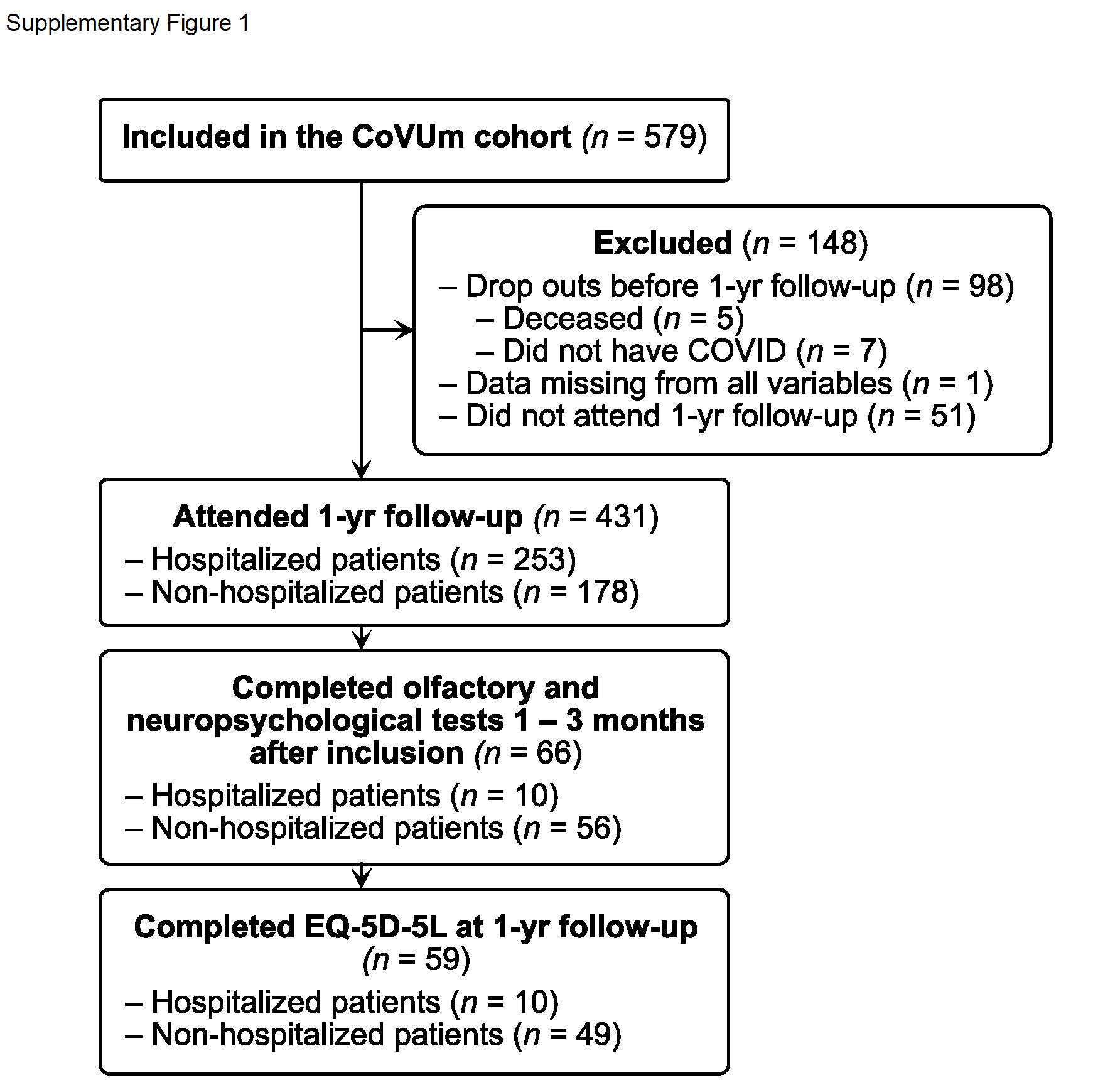

Supplement: Supplementary file 1 — Supplementary Figure 1 Flowchart of participants included in the study cohort. Abbreviations: n, number of patients; EQ‐5D‐5L, EuroQol 5‐dimension 5‐level questionnaire. [file BRB3-14-e3574-s001.tiff]

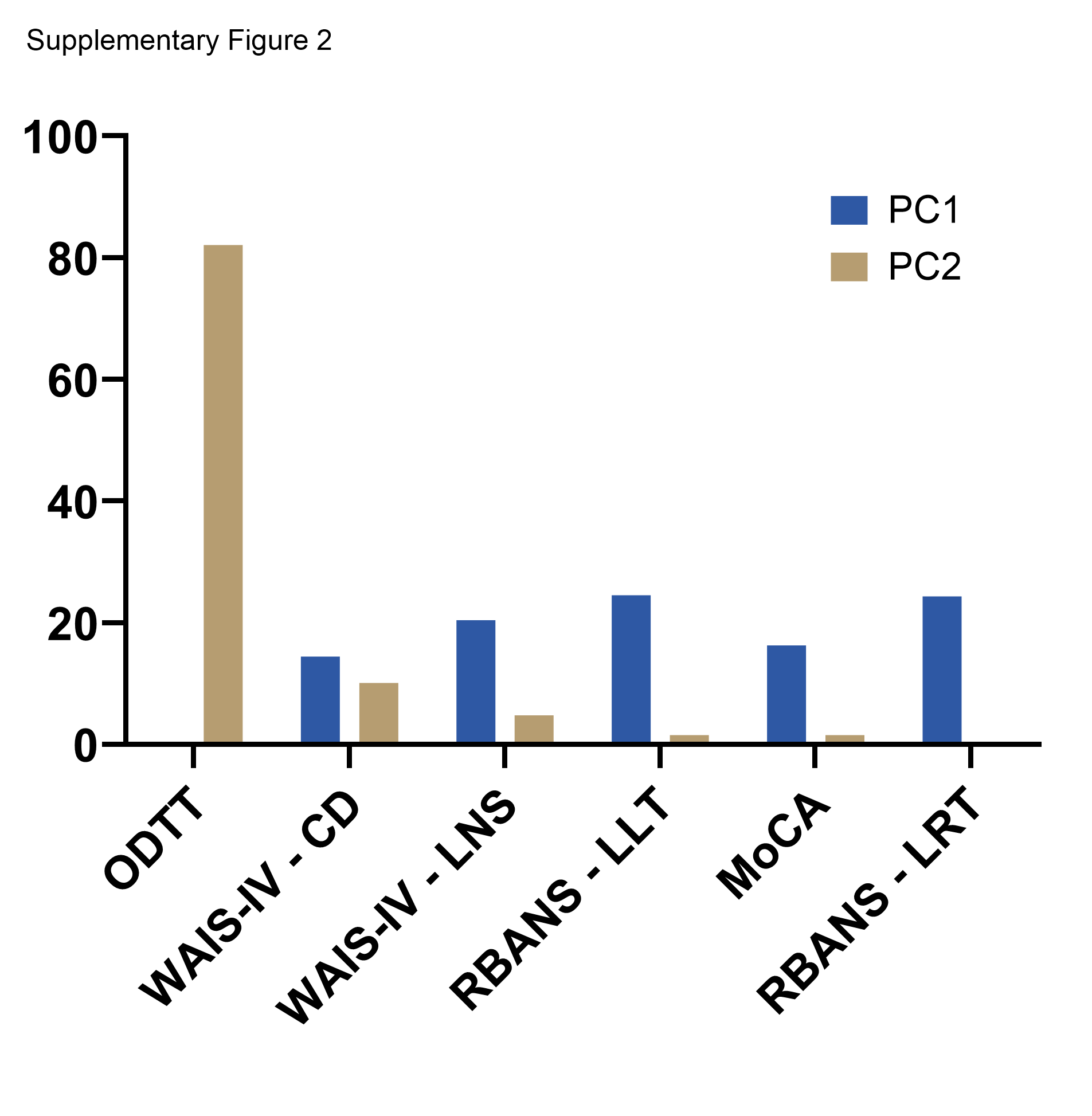

Supplement: Supplementary file 2 — Supplementary Figure 2 The graph represents percentage of contribution for variables in principal component 1 and 2 from the principal component analysis of all neuropsychological and olfactory tests. Abbreviations: PC1, Principal Component 1; PC2, Principal Component 2; ODTT, Olfactory Detection Threshold Test; WAIS‐IV CD, Coding Test from Weschler Adult Intelligence Scale IV; MoCA, Montreal Cognitive Assessment; RBANS—LRT, List recall test from the Repeatable Battery for the Assessment of Neuropsychological Status; RBANS—LLT, List learning test from the Repeatable Battery for the Assessment of Neuropsychological Status; WAIS‐IV LNS, Letter‐Number Sequencing Test from Weschler Adult Intelligence Scale IV. [file BRB3-14-e3574-s002.tif]

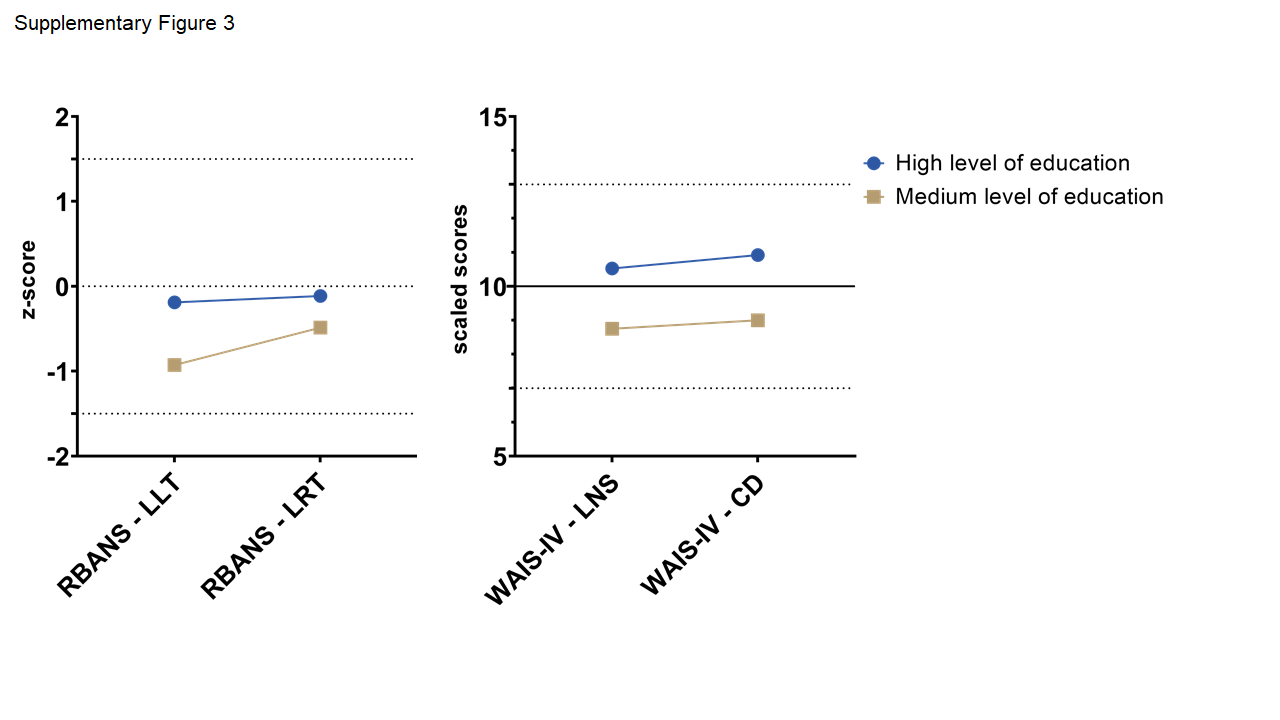

Supplement: Supplementary file 3 — Supplementary Figure 3 Results from neuropsychological assessment, divided by level of education. Abbreviations: RBANS—LLT, List learning test from the Repeatable Battery for the Assessment of Neuropsychological Status; RBANS—LRT, List recall test from the Repeatable Battery for the Assessment of Neuropsychological Status; WAIS‐IV LNS, Letter‐Number Sequencing Test from Weschler Adult Intelligence Scale IV; WAIS‐IV CD, Coding Test from Weschler Adult Intelligence Scale IV. [file BRB3-14-e3574-s003.tiff]
