# Supplementary material for: Olfactory dysfunction as an early predictor for post‐COVID condition at 1‐year follow‐up
Source: Brain Behav. 2024 Jun 6;14(6):e3574. doi: 10.1002/brb3.3574 (PMC11154814; doi:10.1002/brb3.3574)
Supplement: Supplementary file 4 — Supplementary information [file BRB3-14-e3574-s004.docx]

Supplementary information

# Supplementary method

## Olfactory assessment

We developed an olfactory screening method based on the validated and established Connecticut Chemosensory Clinical Research Center Threshold Test (22). The stimulus material consisted of the odorant n-butanol (99.7% VWR) diluted in water to 3.5 mg/m3 and 0.04 mg/m3, corresponding to dilution steps 2 and 6 in Cain (1988). Pure, non-chlorinated and odorless tap water from Umeå was used as blank. Stimuli were presented in 500ml borosilicate glass flasks with poly-propylene screw caps (Simax). Flasks containing the n-butanol dilutions were refrigerated when not in use but taken out two hours prior to testing to reach room temperature. Stimuli were replaced every three days, whereas blanks were changed every day. These materials and have previously been reported to produce almost identical thresholds as those established through professional grade olfactometric procedures (23).

The test comprised a threshold screening part where participants were presented with the stimuli in ascending order (i.e., first dilution step 6, then dilution step 2). They were tasked to sniff and indicate which of two flasks (one with n-butanol, one blank) that contained the odorant, five times in succession. If the patient made an error, the test leader moved on to the next dilution step or ended the threshold test. If the patient correctly selected all five n-butanol flasks at dilution step 6, they were regarded as normosmic. Correct selection of all flasks at dilution step 2 (but failing step 6) indicated hyposmia. Failing to correctly select all dilution step 2 flasks indicated anosmia, as suggested by normative data described in Cain et al. (1988).

The test also included a supra-threshold assessment conducted after the threshold procedure. Participants were tasked to judge the intensity of all flasks (blank, dilution step 6 and 2) using a Borg CR-100 rating scale. The CR-100 has verbal descriptors that correspond to numbers ranging from 0 to 100: Nothing, 0; minimum, 1.5; extremely weak, 2.5; very weak, 6; weak, 12; moderate, 25; strong, 45; very strong, 70; extremely strong, 90; near maximal, 100. Participants were asked for a numerical rating of perceived intensity of the flasks, using the descriptors as anchors. While it is possible to use numbers higher than 100, these are not labelled.

## Cognitive assessment

### The Montreal Cognitive Assessment

The Swedish version of the Montreal Cognitive Assessment (MoCA) was used as a brief screening tool for global cognitive function to detect mild cognitive impairment. MoCA takes approx. The test takes 10 minutes to administer, and it evaluates the following cognitive abilities: executive/visuospatial function, naming, episodic memory, attention, language, abstraction and orientation. Raw scores range between 0 to 30 points, with higher scores reflecting better performance. To correct for education effects one point was added to the total score for participants with twelve years of education or less (if total score < 30p), and <26p was used as a cut off for indicating mild cognitive impairment.

### The Repeatable Battery for the Assessment of Neuropsychological Status

Verbal learning and episodic memory were evaluated with the List learning test (LLT) and the List recall test (LRT) from the Repeatable Battery for the Assessment of Neuropsychological Status (RBANS). Briefly, the list learning test consists of four trials of free immediate recall of a 11-item semantically categorized word list, and the list recall test is a free delayed recall trial of the same wordlist approx. 15 minutes later. The sum of correctly recalled words from the four learning trials (LLT) and the number of correctly recalled words in the delayed recall task (LRT) were used as raw scores and Scandinavian age-corrected normative data were used to generate standardized outcome scores for verbal learning and episodic memory.

### Weschler Adult Intelligence Scale IV

Working memory was assessed with the Letter-Number Sequencing Test (LNS), in which the examiner reads a sequence of random numbers and letters, and then asks the participant to recall first the numbers in ascending order and the letters next in alphabetical order. The length of the sequence is increased at each trial. The test consists of seven items with three trials each, and the number of correct trials is the raw score. Scandinavian, age- corrected norms were used to generate scaled scores with a mean of 10 and a standard deviation of 3.

Speed and attention were evaluated with the Coding test (CD) from WAIS-IV. The CD test uses a key containing nine numbers (1-9), each paired with an abstract symbol and a response sheet consisting of boxes with a number (1-9) in the top of each box, and an empty space below. The participant’s task is to copy the associated symbols to each number and box as rapidly as possible within a 120 second time limit. The number of correctly produced symbols within the time limit was used as raw score and thereafter transformed to Scandinavian age corrected scaled scores with a mean of 10 and a standard deviation of 3.

## Clinical scoring instruments

### The Hospital Anxiety and Depression Scale

Symptoms of depression and anxiety was measured using the Hospital Anxiety and Depression Scale (HADS). 14 items are tested, separated into two subscales, anxiety (HADS-A) and depression (HADS-D). Each item scores from 0 to 3 and each subscale scores range from 0-21. Higher scores indicate more severe symptoms of anxiety or depression. Scores of 8 and above indicates symptoms consistent with depression or anxiety.

### The Multidimensional Fatigue Inventory-20

Fatigue was measured with the Multidimensional Fatigue Inventory-20 (MFI-20). This is a self-report instrument that measures fatigue in five different dimensions; general fatigue, physical fatigue, reduced motivation, reduced activity and mental fatigue. The questionnaire contains four items per dimension, each item scores from 1 to 5. The scale of the separate dimensions scores from 4 to 20, a higher score indicating more fatigue.

### The Karolinska Sleep Questionnaire

The Karolinska Sleep Questionnaire (KSQ) was used to measure subjective sleep and sleepiness. It is a self-report questionnaire that includes 18 questions. The questions can be divided into groups that measures different symptoms: insomnia, wake-ups, snoring and daytime sleepiness. Four questions are intended to assess insomnia, three to assess problems with wake ups during the night as well as snoring and six questions to assess daytime sleepiness. The questions have six levels ranging from 1 = never to 6 = always. Total score in each group was divided by the number of questions in that group to acquire an index. Index ≤ 3 was used as a cut off for indicating problems with a specific symptom.

# Supplementary tables

## Supplementary Table 1.

Demographic and baseline characteristics of the study cohort, divided by initial severity of disease.

|  | **Total  (n = 66)** | **Hospitalised (n = 10)** | **Non-hospitalised (n = 56)** | **p-value** |
| --- | --- | --- | --- | --- |
| **Age in years – median (IQR)** | 51.5 (47.0-59.8) | 55.5 (52.0-59.8) | 51.0 (45.8-59.8) | 0.303ª |
| **Sex – n (%)** |  |  |  |  |
| Women | 26 (39.3) | 5 (50.0) | 21 (37.5) | 0.498ᶜ |
| **BMI – median (IQR)** | 25.6 (23.8-27.4) | 28.5 (25.9-31.8) | 25.4 (23.4-27.2) | **0.033**ª |
| **Comorbidities – n (%)** |  |  |  |  |
| Diabetes | 4 (6.0) | 1 (10.0) | 3 (5.4) | 0.490ᶜ |
| Hypertension | 15 (22.7) | 4 (40.0) | 11 (19.6) | 0.217ᶜ |
| Cardiovascular disease¹ | 5 (7.6) | 0 (0.0) | 5 (8.9) | 1.000ᶜ |
| Chronic lung disease² | 11 (16.7) | 1 (10.0) | 10 (17.9) | 1.000ᶜ |
| Asthma | 10 (15.2) | 1 (10.0) | 9 (16.1) | 1.000ᶜ |
| Autoimmune disease³ | 5 (7.6) | 2 (20.0) | 3 (5.4) | 0.162ᶜ |
| Immunocompromised⁴ | 1 (1.5) | 0 (7.7) | 1 (1.8) | 1.000ᶜ |
| Malignancy⁵ | 1 (1.5) | 0 (0.0) | 1 (1.8) | 1.000ᶜ |
| Brain injury⁶ | 5 (7.6) | 2 (20.0) | 3 (5.4) | 0.162ᶜ |
| Psychiatric illness⁷ | 6 (9.1) | 0 (0.0) | 6 (10.7) | 0.580ᶜ |
| Dyslexia | 3 (4.5) | 0 (0.0) | 3 (5.4) | 1.000ᶜ |
| **CCI – median (IQR)** | 0 (0-0) | 0 (0-0) | 0 (0-0) | 0.104ª |
| **Smoking status – n (%)**⁸ |  |  |  | 0.687ᵇ |
| Non-smoker | 54 (84.4) | 8 (88.9) | 46 (83.6) |  |
| Current smoker | 0 (0.0) | 0 (0.0) | 0 (0) |  |
| Former smoker | 10 (15.6) | 1 (11.1) | 9 (16.4) |  |
| **Level of education – n (%)**⁹ |  |  |  | 0.711ᵇ |
| Lower | 0 (0.0) | 0 (0) | 0 (0) |  |
| Medium | 23 (34.8) | 4 (40.0) | 19 (33.9) |  |
| Higher | 43 (65.1) | 6 (60.0) | 37 (66.1) |  |
| **Other first language – n (%)** | 7 (10.6) | 2 (20.0) | 5 (8.9) | 0.285ᶜ |

¹Ischemic heart disease, congestive heart failure, arrythmias, aortic disease, valvular heart disease or peripheral arterial insufficiency.

² Chronic obstructive pulmonary disease and asthma.

³ Including rheumatic diseases.

⁴ Immune deficiency diseases or immunosuppressive/immunomodulatory medication.

⁵ Solid localized tumor, lymphoma, or leukemia

⁶ Previous brain surgery or head trauma

⁷ History of anxiety, depression or exhaustion syndrome

⁸ Smoking status is missing in 2 patients. The analysis is based on 64 patients.

⁹ Lower: Less than three years beyond Swedish compulsory school. Medium: Three years beyond Swedish compulsory school, but no college or university degree. Higher: University or college degree.

ª Mann-Whitney U-test

ᵇ X²-test

ᶜ Fischer’s exact test

**Abbreviations:** n, number of patients; BMI, Body Mass Index; CCI, Charlson Comorbidities Index; IQR, interquartile range.

## Supplementary Table 2.

Demographic and baseline characteristics of the study cohort, divided by post-covid status at the one-year follow-up visit.

|  | **Total  (n = 59)** | **PCC+ (n = 13)** | **PCC- (n = 46)** | **p-value** |
| --- | --- | --- | --- | --- |
| **Age in years – median (IQR)** | 52 (47-60) | 59 (52.0-60.0) | 51 (46.3-58.8) | 0.105ª |
| **Sex – n (%)** |  |  |  |  |
| Women | 25 (42.4) | 7 (53.8) | 18 (39.1) | 0.362ᶜ |
| **BMI – median (IQR)** | 25.7 (24-27.4) | 28.9 (27.2-33.2) | 25.1 (23.4-26.4) | **<.001**ª |
| **Comorbidities – n (%)** |  |  |  |  |
| Diabetes | 4 (6.8) | 1 (7.7) | 3 (6.5) | 1.000ᶜ |
| Hypertension | 13 (22.0) | 8 (61.5) | 5 (10.9) | **<.001**ᶜ |
| Cardiovascular disease¹ | 5 (8.5) | 2 (15.4) | 3 (6.5) | 0.302ᶜ |
| Chronic lung disease² | 11 (18.6) | 5 (38.5) | 6 (13.0) | 0.053ᶜ |
| Asthma | 10 (16.9) | 4 (30.8) | 6 (13.0) | 0.204ᶜ |
| Autoimmune disease³ | 5 (8.5) | 2 (15.4) | 3 (6.5) | 0.302ᶜ |
| Immunocompromised⁴ | 1 (1.7) | 1 (7.7) | 0 (0) | 0.220ᶜ |
| Malignancy⁵ | 1 (1.7) | 0 (0) | 1 (2.2) | 1.000ᶜ |
| Brain injury⁶ | 5 (8.5) | 2 (15.4) | 3 (6.5) | 0.302ᶜ |
| Psychiatric illness⁷ | 5 (8.5) | 2 (15.4) | 3 (6.5) | 0.302ᶜ |
| Dyslexia | 2 (3.4) | 1 (7.7) | 1 (2.2) | 0.395ᶜ |
| **CCI – median (IQR)** | 0 (0-0) | 0 (0-1) | 0 (0-0) | 0.083 ª |
| **Smoking status – n (%)⁸** |  |  |  | 0.361ᵇ |
| Non-smoker | 50 (84.7) | 10 (76.9) | 40 (88.9) |  |
| Current smoker | 0 (0) | 0 (0.0) | 0 (0) |  |
| Former smoker | 8 (13.6) | 3 (23.1) | 5 (11.1) |  |
| **Level of education – n (%)⁹** |  |  |  | 0.746ᵇ |
| Lower | 0 (0) | 0 (0) | 0 (0) |  |
| Medium | 20 (33.9) | 5 (38.5) | 15 (32.6) |  |
| Higher | 39 (66.1) | 8 (61.5) | 31 (67.4) |  |
| **Hospitalised – n (%)** | 10 (16.9) | 4 (30.8) | 6 (13.0) | 0.204ᶜ |
| **Other first language – n (%)** | 7 (11.9) | 2 (15.4) | 5 (10.9) | 0.643ᶜ |

^1^Ischemic heart disease, congestive heart failure, arrythmias, aortic disease, valvular heart disease or peripheral arterial insufficiency.

² Chronic obstructive pulmonary disease and asthma.

³ Including rheumatic diseases.

⁴ Immune deficiency diseases or immunosuppressive/immunomodulatory medication.

⁵ Solid localized tumor, lymphoma, or leukemia

⁶ Previous brain surgery or head trauma

⁷ History of anxiety, depression or exhaustion syndrome

⁸ Smoking status is missing in 1 patient. The analysis is based on 58 patients.

⁹ Lower: Less than three years beyond Swedish compulsory school. Medium: Three years beyond Swedish compulsory school, but no college or university degree. Higher: University or college degree.

ª Mann-Whitney U-test

ᵇ X²-test

ᶜ Fischer’s exact test

**Abbreviations:** PCC+, Post COVID-19 Condition+; PCC-, Not Post COVID-19 Condition+; n, number of patients; BMI, Body Mass Index; CCI, Charlson Comorbidities Index; IQR, interquartile range.

## Supplementary Table 3.

Health-related quality of life among the study cohort assessed by EQ-5D-5L at the one-year follow-up, divided by sex.

|  | **Total  (n = 59)** | **Woman (n = 25)** | **Men (n = 34)** | **p-value** |
| --- | --- | --- | --- | --- |
| **EQ-5D-5L dimensions – n (%)** |  |  |  |  |
| Problems with mobility | 6 (10.2) | 3 (12.0) | 3 (8.8) | 0.691ᵇ |
| Problems with personal care | 3 (5.1) | 2 (8.0) | 1 (2.9) | 0.569ᵇ |
| Problems with usual activities | 7 (11.9) | 4 (16.0) | 3 (8.8) | 0.443ᵇ |
| Problems with pain or discomfort | 17 (28.8) | 10 (40.0) | 7 (20.6) | 0.147ᵇ |
| Problems with anxiety or depression | 4 (6.8) | 2 (8.0) | 2 (5.9) | 1.000ᵇ |
| **EQ-VAS ≤ 60 – n (%)** | 11 (18.6) | 6 (24.0) | 5 (14.7) | 0.502ᵇ |
| **EQ-VAS - median (IQR)** | 80.0 (70.0-88.0) | 80.0 (65.0-85.0) | 80.0 (70.5-90.0) | 0.430ª |
| **Reduced ability to work – n (%)** | 5 (8.5) | 3 (12.0) | 2 (5.9) | 0.641ᵇ |

ª Mann-Whitney U-test

ᵇ Fischer’s exact test

**Abbreviations:** n, number of patients; EQ-5D-5L, EuroQol 5-dimension 5-level questionnaire, EQ-VAS, EuroQol Visual Analogue Scale.

# Supplementary figure legends

## Supplementary Figure 1.

Flowchart of participants included in the study cohort.

**Abbreviations:** n, number of patients; EQ-5D-5L, EuroQol 5-dimension 5-level questionnaire.

## Supplementary Figure 2.

The graph represents percentage of contribution for variables in principal component 1 and 2 from the principal component analysis of all neuropsychological and olfactory tests.

**Abbreviations:** PC1, Principal Component 1; PC2, Principal Component 2; ODTT, Olfactory Detection

Threshold Test; WAIS-IV CD, Coding Test from Weschler Adult Intelligence Scale IV; MoCA, Montreal Cognitive Assessment; RBANS – LRT, List recall test from the Repeatable Battery for the Assessment of Neuropsychological Status; RBANS – LLT, List learning test from the Repeatable Battery for the Assessment of Neuropsychological Status; WAIS-IV LNS, Letter-Number Sequencing Test from Weschler Adult Intelligence Scale IV.

## Supplementary Figure 3.

Results from neuropsychological assessment, divided by level of education.

**Abbreviations:** RBANS – LLT, List learning test from the Repeatable Battery for the Assessment of Neuropsychological Status; RBANS – LRT, List recall test from the Repeatable Battery for the Assessment of Neuropsychological Status; WAIS-IV LNS, Letter-Number Sequencing Test from Weschler Adult Intelligence Scale IV; WAIS-IV CD, Coding Test from Weschler Adult Intelligence Scale IV.
